# Serum Concentrations of Interleukin-8 and Vitamin D Levels in Jordanian Patients with Rheumatoid Arthritis

**DOI:** 10.2147/JIR.S509565

**Published:** 2025-07-09

**Authors:** Ammar Salim Ali deeb, Seba H Selawi, Kawther Faisal Amawi, Amjad E Hamdallah, Sami Ahmed Zaher Basha

**Affiliations:** 1 Department of Medical Laboratory Sciences, Faculty of Allied Medical Sciences, Zarqa University, Al-Zarqa, Jordon

**Keywords:** autoimmune, rheumatoid arthritis, cytokines, interleukin 8, vitamin D

## Abstract

**Background:**

Rheumatoid arthritis (RA) is a chronic autoimmune disease characterized by persistent inflammation. Vitamin D deficiency and elevated levels of IL-8 have been implicated in the pathogenesis of RA.

**Aims of the Study:**

This cross-sectional study aims to compare Vitamin D and IL-8 levels between RA patients and healthy controls and assess the relationship between Vitamin D, IL-8, and other inflammatory markers in RA patients.

**Methodology:**

The study included 123 participants divided into two groups: 63 RA patients and 60 healthy controls, with equal numbers of males and females in each group. Serum levels of Vitamin D, IL-8, rheumatoid factor (RF), anti-cyclic citrullinated peptide (anti-CCP) antibodies, C-reactive protein (CRP), erythrocyte sedimentation rate (ESR), and white blood cell (WBC) count were measured and compared between the groups.

**Results:**

RA patients had a higher percentage of females (74.6%) compared to the control group (41.7%). Vitamin D levels were significantly lower in RA patients compared to controls (41.4 ± 25.2 vs 46.5 ± 12.4, p = 0.002). IL-8 levels were significantly higher in the RA group compared to controls (546.9 pg/mL vs 3.1 pg/mL, p < 0.001). RF and anti-CCP antibody levels were also significantly higher in RA patients. However, no significant correlation was found between IL-8, Vitamin D, and other inflammatory markers.

**Conclusion:**

The findings highlight the importance of Vitamin D screening or intervention in RA patients due to its potential impact on immune function and disease progression. The elevated IL-8 levels and other inflammatory markers confirm the chronic inflammatory state in RA, emphasizing the need for continuous inflammation-targeted approaches, including pharmacological treatment and lifestyle modifications.

## Introduction

Rheumatoid arthritis (RA) is a chronic autoimmune disease, expressing its inflammatory manifestations within the synovial joints, provoking pain, swelling, and, if untreated, destroying the joints.^1^,^2^ Despite significant progress in the cessation of its physiopathology and the designing of tailored therapies, RA remains difficult for patients and healthcare providers to cope with.^3^,^4^

The complicated interrelation of not only different immune mediators but also of numerous signaling pathways ensures that inflammation is self-maintained in RA.^5^ Of these mediators, IL-8 is the most powerful cytokine that plays the most important role during the inflammatory process in the synovial cavity.^6^ IL-8 is mainly released upon being activated by macrophages, fibroblasts, and endothelial cells in response to the inflammation trigger, and it acts through the production of neutrophils and their activation at sites of inflammation.^7^,^8^

Patients with RA have been found to have elevated levels of IL-8 in joint synovial fluid and the blood was assessed for the disease and joint damage severity.^9^ Along with a wide variety of immune mediators that have been found to be involved in RA, IL-8 and vitamin D have come to light as crucial with the prospects of influencing inflammation responses and dysregulated immunity. IL-8, representing the family of cytokines with a proven pro-inflammatory potency, exhibits its effects by mediating the recruitment and activation of neutrophils in the synovial fluid, whereby inflammation is only amplified in RA patients.^10^,^11^ Besides its aggressive joint damaging effect, the prolonged inflammatory state plays a role in impairing overall health which makes RA chronic disability.^12^

However, vitamin D, which was once known as a bone health and calcium homeostasis regulator, has got the interest of researchers because of its immunomodulatory capacities.^10^ Besides the classical functions, apart from its anti-inflammatory effects, vitamin D is one of the factors that modulate the activities of various immune cells, including T lymphocytes, macrophages, and dendritic cells.^13^ The results of a number of epidemiological studies have shown how low levels of vitamin D in the serum correspond with an increased possibility that a person will relapse or progress into rheumatoid arthritis.^14^ Though the activities of IL-8 and vitamin D in inflammation and immune functioning are quite clear, the exact processes through which they interact in RA are yet to be fully understood. Discovering the interplay between IL-8 and vitamin D concentrations in RA patients might help us to recognize in more depth the complicated immunological interactions that lead to sickness development and progression.

The primary objective of this study is to investigate the correlation between IL-8 and 25-hydroxyvitamin D levels in patients diagnosed with rheumatoid arthritis. To the best of our knowledge, this is the first study to explore the interplay between these biomarkers in Jordanian RA patients.

Therefore, this study aims to compare serum levels of IL-8 and vitamin D between RA patients and healthy controls and assess the relationships between these biomarkers and other inflammatory indicators such as RF, anti-CCP antibodies, CRP, ESR, and WBC count. Understanding these associations may help clarify the roles of IL-8 and vitamin D in RA pathogenesis and guide future diagnostic or therapeutic strategies.

## Subjects and Methods

### Design and Study Population

The current study was carried out between February and April 2024 on 123 participants divided into two groups, 63 patients with RA fulfilling the American Rheumatism Association 1987 revised criteria for the classification of RA and 60 normal healthy age-and sex-matched controls. The study was conducted in a private medical laboratory (Smart Labs, Amman, Jordan). Participants were selected from subjects referred to in the laboratory for routine checkups. This study was conducted in accordance with the principles of the Declaration of Helsinki. Zarqa University approved the study protocol (IRB /ZU/2024/15). The objective of the study was explained, and information sheets and consent forms were distributed to participants before data and blood collection.

Subjects were divided into two groups based on levels of RF. Subjects with RF >14.0 IU/mL were classified as patients, while subjects with lower than these cut-off values were considered controls. History and clinical examinations were conducted. Age, gender, intake of medication, and supplements were assessed as appropriate.

Participants were excluded from the study if they had any of the following conditions: other autoimmune diseases, chronic infections, malignancies, liver or kidney disorders, recent Vitamin D supplementation (within the past three months), corticosteroid or immunosuppressive therapy unrelated to RA, or pregnancy. These criteria were applied to ensure that the observed changes in Vitamin D and IL-8 levels were specifically related to RA and not influenced by other confounding conditions.

### Biochemical Investigations

Ten milliliters of peripheral venous blood was collected from each subject via venipuncture and divided into two parts. The first part was left without an anticoagulant for serum separation, while the second part was collected into sterile EDTA vacutainers for complete blood count and ESR measurements. ESR was recorded in millimeters per hour. CRP, mg/L and RF, IU/mL were analyzed using immunoturbidimetry on a cobas 6000 (Roche) CCP, U/mL was checked using an ELISA assay on a Chorus Trio 2013 system (Italy). Vitamin D (ng/mL) 25-OH vitamin D was evaluated using an enzyme-linked immunosorbent assay in serum samples from patients, on a cobas 6000 (Roche) system.

Total concentrations of IL-8 in serum samples were measured using a commercial Enzyme-linked Immunosorbent Assay (ELISA) Kit (My BioSource, according to manufacturer’s instructions.

### Statistical Analysis

Data were fed to the computer and analyzed using IBM SPSS software package version 20.0. (Armonk, NY: IBM Corp). Qualitative data were described using numbers and percentages. The Kolmogorov–Smirnov test was used to verify the normality of distribution. Quantitative data were described using ranges (minimum and maximum), mean, standard deviation, median, and interquartile range (IQR). The significance of the results obtained was judged at the 5% level.

## Results

### Characteristics of Patients Population

Sixty-three patients with confirmed RA were enrolled in this study. Sixty individuals served as a control group. The details of age and gender of the individual are presented in Table 1. Patients did not receive any treatment. Most of the patients were females (47 females versus 16 males). In controls, there were 35 males versus 25 females. Mean age of RA patients was 44.42 ± 10.01 while that of controls was 36.6 ± 12.9 years.

**Table 1:** Comparison Between the Two Study Groups According to Age and Gender

	Patient (n = 63)	Normal (n = 60)	P*
**Gender**			
Female	47 (74.6%)	25 (41.7%)	<0.001*
Male	16 (25.4%)	35 (58.3%)	
**Age**			
**Mean** ± **SD**	44.42 ± 10.01	36.6 ± 12.9	<0.001*
**Range**	(23–81)	(18–71)	

**Notes**: P*: p-value for comparing between the two study groups. *: Statistically significant at *p ≤ 0.05.*

**Abbreviation**: SD, Standard deviation.

### Serum Levels of IL-8

A statistically significant higher levels of IL-8 (P < 0.001) in RA patients compared to healthy controls, 33.0 (176.5 ± 546.9) versus 2.8 (3.1 ± 2.7) was demonstrated (Table 2).

**Table 2:** Comparison Between the Two Study Groups According to IL-8 and Vitamin D

	Patient (n = 63)	Normal (n = 60)	P*
**Vitamin D (ng/mL)**			
Range.	11.0–146.0	20.0–70.0	0.002*
Mean ± SD.	41.4 ± 25.2	46.5 ± 12.4	
Median (IQR).	34.0 (25.0–47.6)	48.0 (37.0–55.0)	
**IL-8 (pg/mL)**			
Range.	14.0–2870.0	0.1–18.2	<0.001*
Mean ± SD.	176.5 ± 546.9	3.1 ± 2.7	
Median (IQR).	33.0 (26.0–49.0)	2.8 (1.5–4.1)	

**Notes**: P*: p-value for comparing between the two study groups. *Statistically significant at *p ≤ 0.05.*

**Abbreviation**: SD, Standard deviation.

### Serum Level of Vit D

A statistically significant lower levels of Vitamin D (P < 0.001) in RA patients compared to healthy controls, 34.0 (25.0–47.6) versus 48.0 (37.0–55.0) was demonstrated (Table 2).

In Table 3, all inflammatory markers (RF, CCP, CRP, ESR, and WBC) showed significantly higher levels in the patients’ group compared to normal subjects (p= <0.001 for all parameters).

**Table 3:** Comparison Between the Two Study Groups According to Inflammatory Parameters

	Patient (n = 63)	Normal (n = 60)	p
**RF (UI/mL)**			
Range.	14.9–474.2	2.0–11.0	<0.001*
Mean ± SD.	109.7 ± 115.6	6.3 ± 2.4	
Median (IQR)	69.1 (24.0–163.5)	6.0 (4.5–9.0)	
**CCP (U/mL)**	56 (88.9%)	0 (0.0%)	<0.001*
Range.	5.0–300.0	–	–
Mean ± SD.	73.7 ± 97.4	–	
Median (IQR)	25.0 (22.0–62.0)	–	
**CRP (mg/L)**			
Range.	0.2–94.0	0.1–5.0	<0.001*
Mean ± SD.	16.1 ± 23.8	2.0 ± 1.2	
Median (IQR)	6.0 (3.0–15.5)	2.0 (1.0–3.0)	
**ESR (mm)**	43 (68.3%)	60 (100.0%)	<0.001*
Range.	2.0–74.0	2.0–19.0	<0.001*
Mean ± SD.	28.5 ± 19.8	7.2 ± 4.3	
Median (IQR)	26.0 (13.5–39.5)	5.0 (4.0–12.0)	
**WBC (x103 Cells/ µL)**			
Range.	3.4–14.9	4.0–10.6	<0.001*
Mean ± SD.	8.7 ± 3.0	6.7 ± 1.5	
Median (IQR)	8.5 (6.5–11.2)	6.3 (5.4–7.2)	

**Notes**: U: Mann Whitney test. χ^2^: Chi square test. p: p-value for comparing the two studied groups. *Statistically significant at *p ≤ 0.05.*

**Abbreviations**: SD, Standard deviation.

Among the patients’ group, Table 4 showed no correlation between IL-8 and vitamin D with p = 0.737. In addition, Table 5 illustrates that there is no correlation between IL-8 and vitamin D with other inflammatory markers (RF, CCP, CRP, ESR, and WBC) with IL-8 (P= 0.061, 0.387, 0.375, 0.661, and 0.281; respectively), and vitamin D (P= 0.114, 0.416, 0.769, 0.663, and 0.777; respectively).

**Table 4:** Correlation Between IL-8 and Vitamin D in Patients’ Group (n = 63)

	IL-8 (pg./mL)	
	**Rs**	**p**
**Vitamin D**	0.043	0.737

**Abbreviation**: R_s_, Spearman coefficient.

**Table 5:** Correlation Between IL-8 and Vitamin D with Different Parameters in Patient Group

	N	IL-8 (pg./mL)		Vitamin D	
		R_s_	p	r_s_	P
**RF**	**63**	0.238	0.061	−0.201	0.114
**CCP**	**56**	−0.118	0.387	−0.111	0.416
**CRP**	**63**	0.114	0.375	−0.038	0.769
**ESR**	**43**	−0.069	0.661	−0.068	0.663
**WBC (x103)**	**63**	0.138	0.281	−0.036	0.777

**Abbreviation**: Rs, Spearman coefficient.

## Discussion

The current study intended to determine the Plasma Rheumatoid Factor (RF), inflammatory markers, and cytokines among the patient and normal control subjects emphasizing Vitamin D and interleukin-8 (IL-8). This study involved 123 participants divided into two groups: 63 patients and 60 normal subjects, out of each group 15 males and 15 females. To give a clearer perspective on the magnitudes and directions of these outcomes, the results of this study were benchmarked against the results of prior research.

The present study showed that the female patient group had a higher percentage (74. 6%) than the normal group (41.7%). This has been consistent with another study which indicates that autoimmune diseases such as rheumatoid arthritis are more prevalent in the female population compared to the male population (Kvien et al, 2010; Tobón et al, 2010; Cincinelli et al, 2018) showed that female patients account for some 57% of rheumatoid arthritis cases, which is 1.5 times the number of male patients, and this study agreed with that, too. On hormonal differences, especially estrogen, it has been proposed that they contribute to gender differences in autoimmune disease susceptibility.^15–17^

The comparison of the mean vitamin D levels between the patient and the normal group revealed that the patient had lower vitamin D levels than the normal group. As for the patient group the mean Vitamin D level was established to be 41.4 ± 25.2 < w = 46 > 5 ± 12.4. Analyzing the relationship between Vitamin D and autoimmune diseases^18^ identified low levels of the Vitamin as present in individuals with autoimmune diseases and discussed how Vitamin D plays an important role in immune regulation and that deficiency in it increases vulnerability to autoimmune diseases. These findings of a significant difference re-emphasize that Vitamin D supplementation may be effective in the management of rheumatoid arthritis and other inflammatory diseases as an additional therapy.^18^

Vitamin D levels were significantly lower in the RA patient group compared to the controls (p = 0.002). This finding supports previous studies that highlighted the role of Vitamin D deficiency in the pathogenesis and progression of RA. For instance, Rossini et al, 2010; Meena et al, 2018 found that Vitamin D deficiency is common in RA patients and is associated with increased disease activity and severity. Low levels of Vitamin D may contribute to the immunological dysregulation observed in RA, as Vitamin D has immunomodulatory effects that are crucial in maintaining immune homeostasis.^19^,^20^

The levels of IL-8 were significantly higher in the patient group compared to the normal group (MD = 176,95% CI: 121–230; WMD = 0.33,95% CI: 0.21–0.45; p < 0.001). 5 ± 546.9 pg/mL and 3.1 ± 2.7 pg/mL, respectively. Russo et al, 2014 reported that IL-8 exhibits pro-inflammatory properties and has been linked to rheumatoid arthritis due to its contribution to inflammation.^8^ Research has established that IL-8 has a significant role in inflammation, which is associated with rheumatoid arthritis. Physiologically, a rise in the level of IL-8 indicates that there is increased inflammatory activity in the body of patients with rheumatoid arthritis, as established Gremese et al, 2023 who noted that higher levels of IL-8 were found in patients with active rheumatoid arthritis and are associated with disease activity.^9^ The levels of IL-8 were significantly higher in the RA patient group compared to the control group (p < 0.001). The levels of IL-8 were significantly higher in the RA patient group compared to the control group (p < 0.001). IL-8 is a pro-inflammatory cytokine that plays a critical role in the inflammatory process associated with RA. Elevated levels of IL-8 in RA patients have been reported in studies,^21^ indicating its role in promoting the recruitment and activation of neutrophils and other immune cells in the synovial fluid and tissues of RA patients. This study’s findings corroborate these reports, suggesting that IL-8 is a key player in the inflammatory milieu of RA. Both RF and anti-CCP were significantly higher in the patient group or 49 ±13 versus 17±5, p < 0.001, and 32 ±11 versus 8 ± 2, p < 0.001, respectively. This was expected given the persistently elevated inflammatory marker evident in these patients. These findings are also consistent with the data of Nishimura et al, 2007 who observed that high titers of circulating RF and CCP antibodies correlate with rapid disease progression in patients with RA.^22^ CRP and ESR are proven biomarkers of inhabitants and are pragmatic tools in clinical medicine to assess the level of inflammation and response to various treatments.

A cross-sectional comparison of the variables showed no positive relationship between IL-8 and vitamin D in the patient group with a correlation coefficient of 0.737. Additionally, it is worth underlining that there was no graded relationship between IL-8 and other markers of inflammation: RF, CCP, CRP, ESR, and WBC with p-values ranging from 0.061 to 0.661. In the same manner, results on Vitamin D were not different from these inflammatory indicators with corresponding p-values of 0.114 to 0.777. Based on these observations, IL-8 and Vitamin D, have a relationship exclusively with the inflammation process and disease states, but they are unlikely to combine in a way that is reflected in the parameters that were measured in this study. The lack of correlation is in line with Cutolo et al, 2011 where the authors noted that Vitamin D deficiency and inflammatory cytokine levels contribute to disease severity in RA but do not interact with each other.^23^ However, RF and CCP are specific markers for RA, and elevated levels of these markers are associated with more severe disease with an ability to predict disease progression. This study’s results align with previous findings that both RF and CCP are significantly higher in RA patients, underscoring their diagnostic and prognostic value.

Elevated levels of the systemic inflammation markers CRP and ESR in RA patients, as observed in this study, indicate ongoing inflammation and are commonly used to monitor disease activity and response to treatment.

An increased WBC count in RA patients reflects the inflammatory response and is a common finding in active RA. This study’s results are in line with the general observation that RA is associated with leukocytosis due to chronic inflammation. The results of the present study are in almost complete concordance with the earlier findings pointing towards the generally accepted understanding of the disease causation and the following clinical progression of RA and other autoimmune disorders. Several elements have supported the findings such as, the patient group predominantly consisting of females, lower levels of Vitamin D, higher levels of IL-8, and higher significant inflammatory markers have all been documented in the previous literature. Nonetheless, the observed relationship between IL 8 and RA did not show any significant association with Vitamin D or other inflammatory biomolecules; this points to the conclusion that both factors have independent roles to play in the progress of the disease.

The present study has several limitations. Firstly, the number of patient samples is small, which may affect the generalizability of the findings. Secondly, the patients were taking various medications, which could potentially influence the results. Lastly, the use of anti-inflammatory medications among the participants may have affected the inflammatory markers, thereby impacting the study’s outcomes.

## Conclusion

In this study, we found that people with rheumatoid arthritis had higher levels of IL-8 and lower levels of vitamin D compared to healthy individuals. However, these two markers did not show a clear link to each other or to other signs of inflammation. This suggests that IL-8 and vitamin D may affect the disease in different ways. Our findings highlight the importance of checking vitamin D levels in RA patients and suggest that IL-8 could be a useful marker for tracking inflammation. More research is needed to better understand how these factors contribute to the disease.

## Data Availability

Derived data supporting the findings of this study are available from the corresponding author on request.
